# The Need to Look at Transgender and Gender Diverse People’s Health: A Preliminary Descriptive Report on Pain, Sexual Distress, and Health Profile of Five Transmasculine People and One Non-Binary Person with Endometriosis

**DOI:** 10.3390/healthcare12121229

**Published:** 2024-06-20

**Authors:** Sérgio A. Carvalho, Teresa Lapa, Patrícia M. Pascoal

**Affiliations:** 1 University of Coimbra, Center for Research in Neuropsychology and Cognitive Behavioral Intervention (CINEICC), 3000-115 Coimbra, Portugal; sergiocarvalho@fpce.uc.pt; 2 Anesthesiology Departament, Hospitais da Universidade de Coimbra, 3004-561 Coimbra, Portugal; teresalapa@hotmail.com; 3 Faculty of Health Sciences, Universidade da Beira Interior, 6200-506 Covilhã, Portugal; 4 Lusófona University, HEI-Lab: Digital Human-Environment Interaction Labs, 1700-097 Lisbon, Portugal

**Keywords:** transgender and gender diverse, endometriosis, healthcare equity, sexual distress, pain

## Abstract

The sexual health of transgender and gender diverse (TGD) people with endometriosis has been overlooked, and important emotional experiences, such as sexual distress and its correlates, have been ignored. This has prevented a more comprehensive look at the health experiences of TGD individuals. This descriptive online survey study preliminarily explored the experiences of pain symptoms, sexual distress, and mental health of N = 6 TGD individuals diagnosed with endometriosis. Descriptive results showed a mean delay of 10 years from the onset of symptoms to the diagnosis. Endometriosis-related pain was a common symptom, although with low to moderate intensity. Results also showed higher mean levels of pain impact, powerlessness and lack of control, somatization, depression, anxiety, and sexual distress, and lower mean levels of emotional well-being, social support, and worse self-image compared to reports on cisgender women with endometriosis in the literature. These results suggested that sexual and mental health in the context of TGD people with endometriosis has specificities and may be associated with factors that need to be accounted for to provide comprehensive and socially just healthcare, such as the recognition of the impact of endometriosis treatment on symptoms of gender dysphoria. To achieve sexual health equity for TGD people, continuous and updated professional training and inclusive research with multiple informants are necessary.

## 1. Introduction

### 1.1. Endometriosis in Trans Masculine Individuals

Endometriosis is a gynecological condition characterized by the presence of ectopic endometrial tissue that can be associated with persistent pain (menstrual pelvic pain as well as acyclical non-menstrual pain) [1,2], with a great detrimental impact on mental health [3] and on sexual health [4]. The prevalence of endometriosis is presented in the literature, varying from 1% to 5% in more recent systematic reviews [5] and 10% in classic prevalence studies [6], depending on the study design and case definition. However, most studies on endometriosis have been conducted in samples of cisgender women, i.e., women with female sex assigned at birth, with very few studies exploring the prevalence, presentation, and impact of endometriosis in individuals assigned female sex at birth who identify as transgender or gender diverse (TGD), i.e., individuals whose gender identity differs from the gender socially attributed to their sex assigned at birth [7]. Those who did so suggest that TGD individuals present an approximate 25% prevalence of endometriosis [7,8].

Although not yet empirically tested, some authors have suggested that trans masculine individuals are at a higher risk of developing endometriosis because of the impact of long-term testosterone therapy—for example, due to heightened estrogenic activity resulting from elevated androgen levels, which is hypothesized to result from the conversion of exogenous testosterone to estradiol in peripheral tissues [9]. One recent study found that the prevalence of pelvic pain, which is a common symptom of endometriosis, in trans masculine individuals increased after testosterone treatment in 71 of the total sample (N = 280), and those experiencing pelvic pain were also more likely to have a prior history of endometriosis [10]. Hence, it is crucial to explore endometriosis in trans masculine individuals further, given that testosterone therapy commonly used by trans masculine individuals is hypothesized to impact endometriosis symptoms in this population [11,12]. Additionally, more research is needed to understand the relationship between individual health profiles and the short- versus long-term impact on the endometrial pathology of gender-affirming hormones [13,14]. Further examining the experience of TGD individuals with endometriosis is of crucial importance to conducting trans-specialized healthcare provision, such as specialized knowledge on the potential impact of hormone therapy on endometriosis, which should guide the medical decision-making process and the development of a treatment plan that successfully balances gender-affirming care and mitigation of endometriosis symptoms [15] given that their experience in the context of endometriosis healthcare seems to be of medical gaslighting and discrimination [16].

### 1.2. Sexual Health and Endometriosis in Trans Masculine Individuals

Sexual health is defined by the World Health Organization (WHO) as a state of physical, emotional, mental, and social well-being related to sexuality and not merely the absence of sexuality-related illness and/or dysfunction (see Edwards and Coleman [17] for a historical overview). Sexual dysfunction occurs when impaired sexual functioning is accompanied by sexual distress, which can be determined by the interaction of multiple factors (e.g., biological, socioeconomic, religious, political, cultural) [18]. Mental health challenges (e.g., symptoms of depression and anxiety) can suppress desire and arousal, as well as the ability to orgasm [19], and are commonly associated with sexual dysfunctions [20]. On a psychological level, low body satisfaction and a negative self-image—a common experience in endometriosis [21,22]—have a reported impact on sexual distress and/or functioning in cisgender women [23], including in women with endometriosis [24,25], whose sexual distress seems to be neither a direct nor exclusive consequence of dyspareunia (i.e., pain during intercourse) [26]. However, few studies have examined the sexual distress of TGD people. In fact, the literature lacks a systematized description of TGD sexual health, which should include a comprehensive and inclusive assessment of multiple impactful factors such as social determinants of health (e.g., access to TGD-specialized healthcare) [27].

TGD individuals experience sexual distress [28], even though sexual desire and arousal seem to be positively impacted by gender-affirming medical interventions, such as hormone therapy, in trans masculine individuals (e.g., [27]). Nonetheless, research on trans masculine individuals’ sexual health is incipient and highly focused on sexual risk behaviors and sexually transmitted diseases (e.g., [28,29]). Also, the scope of some of the existing research overfocuses on sexual function [30], even in studies that aim to examine sexual pleasure—for example, by measuring “pleasure” as the capacity to experience orgasm [31]. Even less is known about gender non-binary individuals’ sexual health, whose sexual functioning might exist outside the existing gender binary conceptualizations of sexual functioning, hence calling for the increase of participant-centered research and patient-centered care [32]. The relationship between pain (a symptom common in patients with endometriosis) and sexual distress and/or functioning is mainly studied in trans masculine individuals in the context of the impact of implanted penile prostheses [33], rather than by exploring pain symptoms as overall presentations of chronic illness, with the impact on sexual distress not necessarily nor exclusively related to painful sexual intercourse.

### 1.3. Aims 

With the expansion of reproductive medicine to include TGD people’s reproductive aspirations, more research must be conducted to assess better, diagnose, and provide specialized healthcare to TGD individuals with endometriosis [34]. TGD individuals with endometriosis may encounter healthcare systems that might overlook their risk for endometriosis, as this is viewed as inherently a “women’s issue”. The current preliminary study aimed to explore the extent of pain symptoms, sexual distress, mental health, and health profile of N = 6 TGD individuals diagnosed with endometriosis, thus contributing to a better understanding of the health specificities of TGD individuals suffering from endometriosis, and, in the long run, for more inclusive healthcare provision for TGD individuals [35]. 

## 2. Materials and Methods

### 2.1. Participants

The current study was conducted in the context of a more extensive three-wave longitudinal study (ongoing) that aims to explore the biopsychosocial factors underlying the relationship between pain, endometriosis symptoms, sexual distress, and mental health of people living with endometriosis. Eligibility criteria included (a) female sex assigned at birth (with any gender identity); (b) more than 18 years of age; (c) with an endometriosis diagnosis conducted by a medical doctor specialized in Obstetrics and Gynecology; (d) with Portuguese nationality; (e) not being currently pregnant. Of the 248 people who accessed the online questionnaire, 230 have completed the study’s assessment protocol. Although the more extensive study did not aim to specifically collect data on TGD individuals with endometriosis, the protocol was completed by six TGD individuals with endometriosis (given that the more extensive study did not aim to study exclusively cisgender women and we anticipated that transgender people could participate, sex assigned at birth and gender identity was asked in the sociodemographic section of the protocol), thus providing a valuable opportunity to preliminarily explore their health profile. 

The current sample presented a mean age of *M* = 33.5 (*SD* = 7.71; Min = 26; Max = 48). Regarding gender identity, five reported being transmasculine, while one identified as non-binary. Regarding marital status, five participants were single, and one was married or in a civil union. Three participants reported having completed high school, two reported having a bachelor’s degree, and one reported having a master’s degree. Regarding occupation, one participant reported being an information technology specialist, one a patient service assistant (hospital staff), two kindergarten teacher’s assistants, one student, and one reported being on sick leave without reporting their occupation. Regarding annual gross income, one participant reported earning between EUR 0–5000, three between EUR 10,001–13,500, one between EUR 13,501–17,000, and one between EUR 17,001–27,000. 

### 2.2. Procedure

The current study was conducted according to the ethical and deontological guidelines and principles in the Helsinki Declaration [36] and those in the European Textbook on Ethics in Research [37]. The Ethics and Deontology Committee of the Faculty of Psychology and Educational Sciences of the University of Coimbra evaluated and approved the ethical procedures and methodology of the current study. The current study was conducted with the help of Portuguese associations of patients with endometriosis, which advertised the study and the online protocol, as well as through social media pages (e.g., groups of people with endometriosis). The endometriosis associations that agreed to collaborate with the study are non-lucrative associations that do not provide medical/clinical treatment but are rather spaces led by people with endometriosis in which informal advice and information on news and scientific advances on endometriosis are provided. Participants responded to an online protocol composed of sociodemographics, medical history, and questionnaires aiming to explore the more extensive study’s scientific questions. Data were collected through an online platform (Limesurvey) allocated to the university server to which the lead author is affiliated. Participants provided informed consent, which ensured (a) the voluntary nature of participation; (b) the confidentiality of data; (c) that only anonymized data would be disseminated; (d) that all datasets would be protected with a password; and (e) only the three researchers directly involved in the study would have access to datasets. No compensation was given for participating in the study.

### 2.3. Instruments

#### 2.3.1. Endometriosis Health Profile (EHP-30)

This measure (original study: Jones et al., 2001; Portuguese validation: Nogueira-Silva et al., 2015) [38,39] is currently the most used self-report measure of health-related quality of life in endometriosis, composed of thirty items that assess five health-impacting dimensions of quality of life in endometriosis: pain impact (e.g., “Been unable to do jobs around the home because of the pain?”), perceived control and powerlessness (e.g., “Felt frustrated because your symptoms are not getting better?”), emotional well-being (e.g., “Had mood swings?”), perceived social support (e.g., “Felt others do not understand what you are going through?”), and self-image (e.g., “Felt your appearance has been affected?”). Items are ranked using a 5-point Likert scale from *never* (0) to *always* (4), where higher scores mean a worse endometriosis-related quality of life. To our knowledge, the EHP-30 has not been psychometrically validated for TGD individuals. The original study found good to excellent internal consistency (from α = 0.83 α = 0.93), as well as the validated Portuguese version (from α = 0.88 to α = 0.98) [39]. 

#### 2.3.2. Sexual Distress Scale-Revised (SDS-R)

The SDS-R (Original study: Derogatis et al., 2008; Portuguese validation: Tavares et al., 2022) [40,41] is the revised version of the female sexual distress scale with an additional item that assesses low sexual desire (“Are you bothered by low sexual desire?”). Although this measure was developed to assess female sexual distress, it has been successfully used and is a psychometrically valid tool to assess sexual distress in men [42]. It is composed of 13 items that assess sexual distress one-dimensionally on a 5-point Likert scale from *never* (0) to *always* (4), where higher scores mean higher sexual distress. To our knowledge, the SDS-R has not been psychometrically validated for TGD individuals. The original validation study of SDS-R in men found excellent internal consistency for both sexually functional (α = 0.94) and sexually dysfunctional (i.e., experiencing sexual difficulties in the last three months and receiving treatment) men (α = 0.93) (Santos-Iglesias et al., 2018 [42]). In the Portuguese validation study, internal consistency was also excellent in the sample of men (ω = 0.92) [41]. 

#### 2.3.3. Brief Symptom Inventory-18 (BSI-18)

This measure (original study: Derogatis, 2001; Portuguese validation: Nazaré et al., 2017) [43,44] is an 18-item scale that assesses psychological distress in the last week on a 5-point Likert scale from *not at all* (0) to *extremely* (4). The measure assesses psychological distress according to three factors of psychosymptomatology: somatization (e.g., “Pains in heart or chest”), depression (e.g., “Feeling no interest in things”), and anxiety (e.g., “Feeling tense or keyed up”); higher scores indicate higher levels of somatization, depression, and anxiety. To our knowledge, the BSI-18 has not been psychometrically validated for TGD individuals. The original study found good values of internal consistency for somatization (α = 0.74), depression (α = 0.84), and anxiety (α = 0.79) [44], as well as the Portuguese validation study (α = 0.80, α = 0.86, α = 0.80, respectively) [43]. 

### 2.4. Data Analyses

All statistical procedures were conducted using SPSS software (version 27). Preliminary analyses were conducted to assess the normality of the data distribution. The normality of data distribution was assessed through the Shapiro–Wilk test, where *p* > 0.05 indicated no severe violations of normality [45]. Descriptive statistics were conducted to explore levels of endometriosis quality of life, sexual distress, and psychological distress according to central tendency measures (i.e., Mean, Standard Deviation). The interpretation of descriptive results was conducted by comparing results with other population-equivalent studies (i.e., Nogueira-Silva et al., 2015 for results of EHP-30; Santos-Iglesias et al., 2018 for results of SDS-R; Nazaré et al., 2017 for results of BSI-18) [39,43,46]. 

## 3. Results

### 3.1. Preliminary Analyses

Preliminary analyses assessing data adequacy, namely normality, and outliers, revealed no severe violation of normality. The Shapiro–Wilk test showed the normal distribution of data (i.e., all variables with *W* between 0.86 and 0.98, and *p*-value between 0.206 and 0.928). No extreme nor moderate univariate outliers were found. No missing data was found, given that the online protocol required all items to be filled in for it to be successfully submitted. 

### 3.2. Descriptive Analyses

Results showed that the participants’ mean age when the first endometriosis symptom(s) occurred in our sample was *M* = 18.3 (*SD* = 5.2; Min = 12; Max = 24), and the mean age for first seeking medical treatment for these symptoms was *M* = 18.7 (*SD* = 9.6; Min = 17; Max = 25). Participants had a mean age of *M* = 28.0 (*SD* = 4.82; Min = 24; Max = 35) when the endometriosis diagnosis was provided. Regarding endometriosis symptoms, all participants (N = 6) reported experiencing dysmenorrhea, pain during and/or after sex, and bloating, while five participants reported having non-menstrual pelvic pain, pain during ovulation, and fatigue. Two participants reported pain during evacuation, and one reported bladder pain. Two participants reported experiencing pain daily, two weekly, and two monthly. Using a numeric pain rating item (0 = no pain; 10 = worst possible pain), one participant reported experiencing moderate levels of pain intensity (4.3) in the last 24 hours, while the other five participants reported low levels of pain intensity (between 0.0 and 1.7). 

In terms of endometriosis treatments, all participants reported not having gone through a hysterectomy, but four participants reported having had one surgery. All participants are currently in endometriosis-related hormone therapy (i.e., hormonal contraceptives for systemic use), and five participants report using analgesia medication: five reported using nonsteroidal anti-inflammatory drugs, three reported using antipyretic analgesics, one reported using opiate (narcotic) analgesics, and one reported using antispasmodics. One participant reported having resorted to physiotherapy to treat endometriosis-related pelvic pain. Besides endometriosis-related pain, two participants reported having other chronic pain diagnoses: one (trans man) reported having fibromyalgia, and one (gender non-binary person) reported having migraine and irritable bowel syndrome. 

Regarding sexual health, two participants (both trans men) reported experiencing sexual problems due to endometriosis-related pain: one participant (age 26) reported “*pain during penetration*”, while another participant (age 32) described “*I have the muscles too stiff, which causes pain. I cannot experience pleasure; my clitoris is dead*”. Three participants reported not experiencing sexual problems, while one reported, “*I prefer not to answer*”. 

Table 1 depicts means and standard deviations on endometriosis-related health profile, sexual distress, and psychological suffering. In terms of the health impact of endometriosis, participants presented a mean value of *M* = 43.2 (*SD* = 31.2) for endometriosis-related pain impact, *M* = 52.1 (*SD* = 18.6) for feelings of lack of control and powerlessness, *M* = 43.8 (*SD* = 19.8) for reduced emotional well-being, *M* = 55.2 (*SD* = 29.4) of reduced social support, and *M* = 72.2 (*SD* = 26.7) of negative self-image. Regarding sexual distress, participants had an *M* = 32.5 (*SD* = 13.0). In terms of mental health, participants reported a *M* = 8.3 (*SD* = 2.5) for symptoms of somatization, *M* = 7.7 (*SD* = 1.8) for depressive symptoms, and *M* = 8.5 (*SD* = 2.9) for anxiety symptoms. 

## 4. Discussion

Although TGD individuals assigned female sex at birth can have endometriosis, knowledge of the specificities of endometriosis in this population is limited [11,12]. The current preliminary study sought to explore the (mental) health profile of six TGD individuals diagnosed with endometriosis by examining the endometriosis-related medical profile of six TGD individuals, as well as their mental health (symptoms of somatization, depression, and anxiety) and sexual distress. 

Our results suggest that there is a mean delay of approximately 10 years between the beginning of endometriosis symptoms (mean age of 18.3) and the actual endometriosis diagnosis (mean age of 28.0), which is not explained by the time lag between the first symptoms and the first healthcare seeking (mean age of 18.7). This delay in diagnosis seems to be higher than that of cisgender women with endometriosis: cisgender women aged 18–45 years present a mean of 6.7 years of time-lag from first symptoms to endometriosis diagnosis [47]. This higher delay may be explained by the lack of knowledge of endometriosis symptom presentations by medical professionals, who may disregard TGD individuals’ symptomatology as clinically relevant and/or attribute endometriosis struggles to gender dysphoria [16]. It could also be the case that TGD individuals with endometriosis may avoid gynecological clinical settings due to fear of discrimination and/or gender dysphoric experiences. 

Results suggest that pain was a common symptom, with all participants (N = 6) reporting experiencing dysmenorrhea (i.e., pain during the menstrual cycle) and pain during and/or after sex, and almost all (*n* = 5) reporting non-menstrual pelvic pain and pain during ovulation. Although pain was common and frequent, pain intensity in the last 24 hours was reported as low by almost all participants (*n* = 5), which may be explained by their use of endometriosis-related hormone therapy and analgesia medication. It is worth mentioning that two participants reported having other chronic pain conditions (i.e., fibromyalgia, migraine, and irritable bowel syndrome), which does not allow us to definitively conclude whether their responses to the numeric pain rating regarded endometriosis-related pain or pain-related to other chronic pain diagnoses. Nonetheless, given that endometriosis-related pain was reported as frequent by all participants, but its intensity was perceived as low, this might suggest that more than pain intensity itself, the impact of pain should be empirically explored in its psychosocial dimension. This is in line with the literature on chronic pain, which is acknowledged to be a multidetermined phenomenon not reduced to the sensory nociception of pain, but rather an inherently cognitive-emotional experience [48]. The psychological elements inherent to pain are increasingly acknowledged in endometriosis, calling for a biopsychosocial approach to endometriosis pain [49]. 

Our sample reported higher levels of pain impact, higher levels of powerlessness and lack of control, lower emotional well-being, lower social support, and worse self-image comparatively to a sample of Portuguese cisgender women with endometriosis (respectively: *M* = 37.1, *SD* = 30.3; *M* = 40.9, *SD* = 30.4; *M* = 41.2, *SD* = 27.4; *M* = 40.2, *SD* = 29.2; *M* = 34.1, *SD* = 27.6) [39]. These results might be influenced by the intersection of the biological specificities of endometriosis in TGD people (e.g., the impact of gender-affirming hormone therapy on androgen levels, contributing to changes in symptom presentation) [9], as well as the compounding minority-related factors contributing to the social determinants of health. Although studies point out that TGD individuals develop resilience and resort to protective resources in the face of gender-related minority stressors (e.g., discrimination, harassment) [50], family rejection [51], and social discrimination, including in healthcare settings [52], these stressors are still experienced by TGD individuals. These gender-related minority specificities might contribute to lower levels of perceived control and higher powerlessness, lower social support, and lower emotional well-being. Results on the impact of endometriosis on self-image were those with the most striking difference from our reference sample of Portuguese cisgender women with endometriosis [39], where our sample presented almost three times the impact of endometriosis on self-image. One possible explanation is that endometriosis symptoms and treatment, due to their being inherently gynecological, may increase symptoms of gender dysphoria, thus contributing to a negative self-image that intersects with the impact of endometriosis itself [53]. 

Our sample presented higher mean levels of somatization, depression, and anxiety compared to a community sample of Portuguese individuals (respectively: *M* = 3.7, *SD* = 4.0; *M* = 4.0, *SD* = 4.2; *M* = 4.9, *SD* = 4.1) [43]. This result might reflect the compounding negative impact of both endometriosis and the gender-related minority stress TGD individuals might experience [54]. Mounting evidence suggests that the social discrimination experienced by TGD individuals has a significant impact on their mental health [55,56] due to their multi-level ecological impact (i.e., intrapersonal, interpersonal, and structural) [57]. Our results on psychopathological symptoms in TGD individuals with endometriosis should be interpreted through both a biopsychosocial and affirming lens, considering both the relationship between minority stress and endometriosis-relevant physiological phenomena (e.g., inflammatory processes) [58], as well as the relationship between minority stress and mental health vulnerability [59]. 

Additionally, our sample reported higher mean levels of sexual distress comparatively to a sample of cisgender sexually dysfunctional men (i.e., experiencing sexual difficulties in the last three months and receiving treatment; *M* = 26.2, *SD* = 11.3) [42]. The sexual health of trans masculine individuals should be interpreted in light of a complex relationship of factors, such as cognitive-affective factors (e.g., fear of damaging organs post-surgery), physiological impact of hormone therapy (e.g., vaginal dryness, hypersensitivity of enlarged clitoris), distal minority stress factors (e.g., the impact of healthcare discrimination and/or reduced access to trans-specialized care), and proximal minority stress factors (e.g., internalized stigma, which can affect the impact of gender congruence on sexual satisfaction) (e.g., [18,60,61]). We do not have information on which gender-affirming medical care the participants in our sample had access to; thus, an in-depth analysis of the biopsychosocial factors contributing to the reported elevated sexual distress is unwarranted. However, two participants reported experiencing sexual problems, namely pain during penetration and difficulties in experiencing sexual pleasure. The fact that only one participant reported pain-related sexual problems, while the sample presented a higher mean level of sexual distress, calls for a more nuanced analysis of the factors contributing to sexual distress in TGD individuals with endometriosis. It should also be noted that not all trans masculine individuals engage in penetrative sex [62,63], thus the impact of pain during sex should not be the only criterion to assess the impact of pain on sexual distress, especially in TGD with endometriosis—which pain is not exclusively dyspareunia. Pain is a complex biopsychosocial phenomenon, which suggests the need to conduct multi-level interpretations of the relationship between pain, sexual distress, and social factors, such as social support. This is not only aligned with the literature on the psychosocial dimension of pain [64], as it also potentially adds a layer of trans-specificity regarding the low social support as a potential result of minority-related stressors (e.g., family rejection, social isolation, healthcare discrimination) [51,52]. In addition, other factors can contribute to sexual distress. For example, empirical findings suggest that cisgender women with endometriosis may experience sexual distress due to a negative body self-image [25], which seems to be in line with the higher mean levels of endometriosis-related negative body self-image in the current sample.

The results of this exploratory study should not be read without considering its methodological limitations. First, our small sample size (*n* = 6) does not allow for a more complex and powered statistical analysis that conclusively provides evidence of the relationship between variables. Future studies should collect a larger sample to conduct more conclusive statistical analyses. Also, this study did not follow probabilistic sampling, so our sample of TGD individuals does not represent the TGD population. Even though some participants reported having sexual problems, the measure used to assess sexual distress was not developed to explore specific sources of sexual distress in TGD individuals; therefore, it is impossible to determine the exact source of sexual distress experienced by the participants. It should also be noted that the current study is embedded in a larger one that does not focus on endometriosis in TGD individuals, and the study was not advertised as such; thus, self-selection bias might have occurred (for example, trans masculine individuals for whom endometriosis is a trigger of dysphoric symptoms might have self-excluded from the study). Future studies should specifically target TGD individuals with endometriosis, thus potentially collecting a more representative sample and exploring the impact that the delay between first symptoms and diagnosis may have on the progression of endometriosis. Future studies need to understand better the intersection between TGD endometriosis status and characteristics and important sexual outcomes such as orgasm, pleasure, and distress related to sexual function, to understand if these aspects are routinely assessed in the TGD population, and in what ways sexual healthcare related to the sexual function of TGD with endometriosis can be improved. In line with this, although an all-encompassing measure of sexual distress allows for the assessment of sexual distress in samples of different gender identities, it has the limitation of lacking trans-specificity. A new measure specifically designed to assess sexual distress related to sexual function in TGD people should be developed to better grasp the specificities of distressful sexual functioning in this population. Finally, it is crucial that future studies with multiple informants (e.g., healthcare providers; TGD people) further explore and take a comprehensive look at factors that may delay proper diagnosis and healthcare related to endometriosis in TGD people. Using qualitative methodologies may be adequate to better understand the individual and systemic processes behind TGD healthcare in the context of endometriosis.

## 5. Conclusions

Although the methodological limitations of the study, namely its small sample size (N = 6), do not warrant generalizing these findings, the results tentatively suggest that TGD people with endometriosis may experience worse mental health challenges, more constraints in sexual function, diminished experiences of pleasure, and augmented sexual distress compared to cisgender people. This may result from a complex relationship between general endometriosis factors (e.g., endometriosis-related symptoms) and minority stressors (e.g., objective discrimination within healthcare provision; endometriosis symptoms as triggers of symptoms of gender dysphoria). Healthcare professionals must acquire adequate and specialized knowledge of TGD health needs to provide socially just and inclusive healthcare. This study highlights the need to further study the mental, physical, and sexual health of TGD individuals with endometriosis and calls for an increase in the visibility of TGD health in behavioral and sexual medicine. 

## Figures and Tables

**Table 1:** Means, standard deviations, and range scores (N = 6).

Variable	*M*	*SD*	Range-Score (Min-Max)
1. Pain (EHP-30)	43.2	31.2	0.0–93.2
2. Control and powerlessness (EHP-30)	52.1	18.6	33.3–75.0
3. Emotional well-being (EHP-30)	43.8	19.8	16.7–70.8
4. Social support (EHP-30)	55.2	29.4	18.8–93.8
5. Self-image (EHP-30)	72.2	26.7	33.3–100.0
6. Sexual distress (SDS-R)	32.5	13.0	12.0–45.0
7. Somatization (BSI-18)	8.3	2.5	5.0–12.0
8. Depression (BSI-18)	7.7	1.8	5.0–10.0
9. Anxiety (BSI-18)	8.5	2.9	5.0–12.0

Note. EHP-30 = Endometriosis Health Profile; SDS-R = Sexual Distress Scale-Revised; BSI-18 = Brief Symptom Inventory.

## Data Availability

Anonymized data, without identifiable sociodemographic data, will be made available upon reasonable request.
